# Supplementary material for: Inhibition of the Growth of Breast Cancer-Associated Brain Tumors by the Osteocyte-Derived Conditioned Medium
Source: Cancers (Basel). 2021 Mar 3;13(5):1061. doi: 10.3390/cancers13051061 (PMC7959137; doi:10.3390/cancers13051061)
Supplement: Supplementary file 1 [file cancers-13-01061-s001.zip › Supplementary Materials/cancers-1088149-original WB figures.pdf]

Uncropped gels

Figure 1

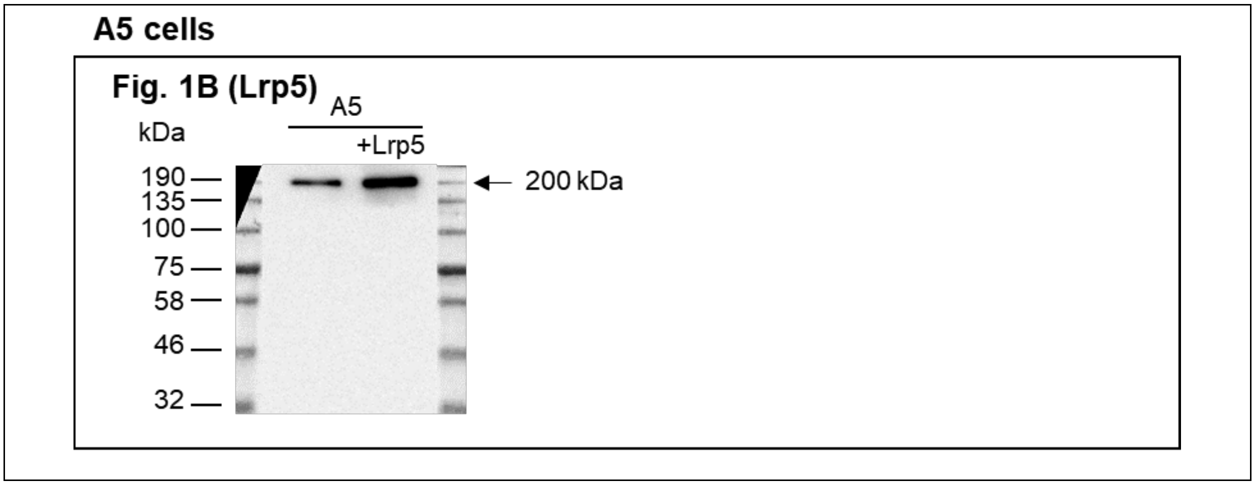

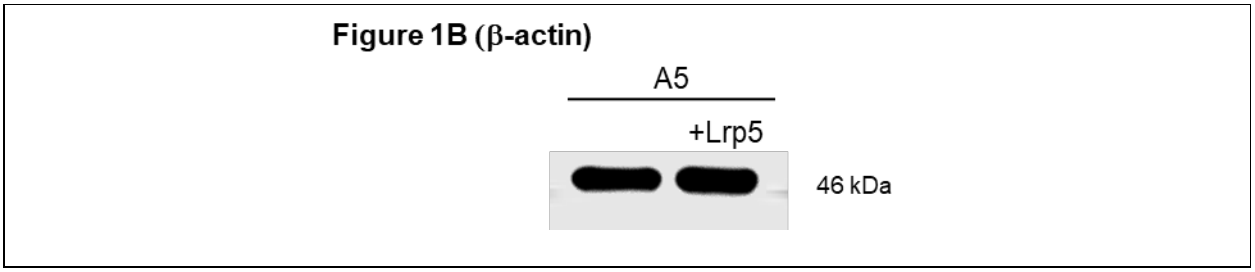

Figure 4

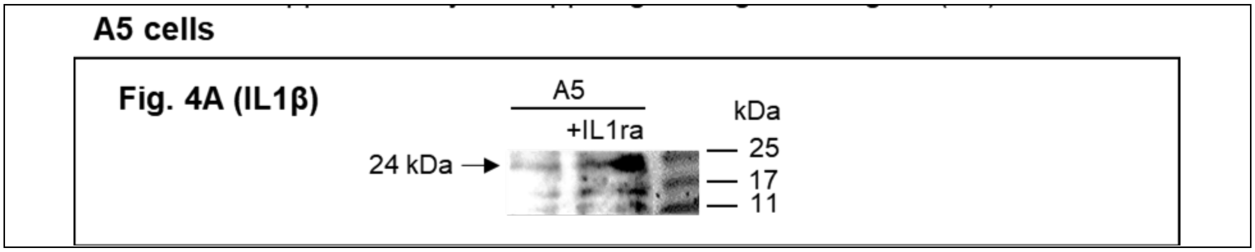

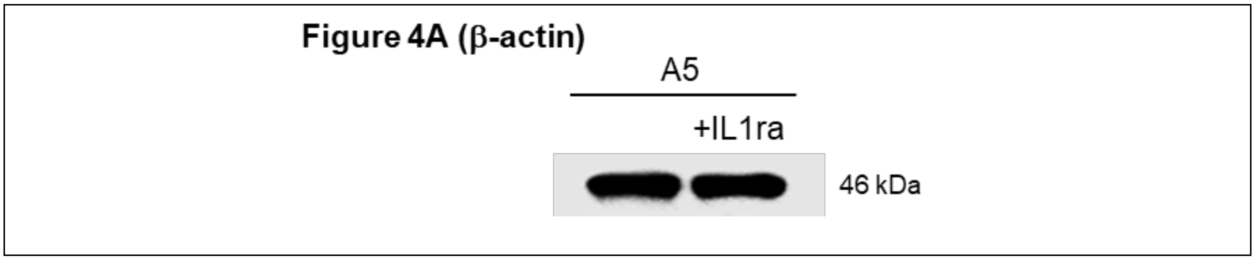

Figure 6

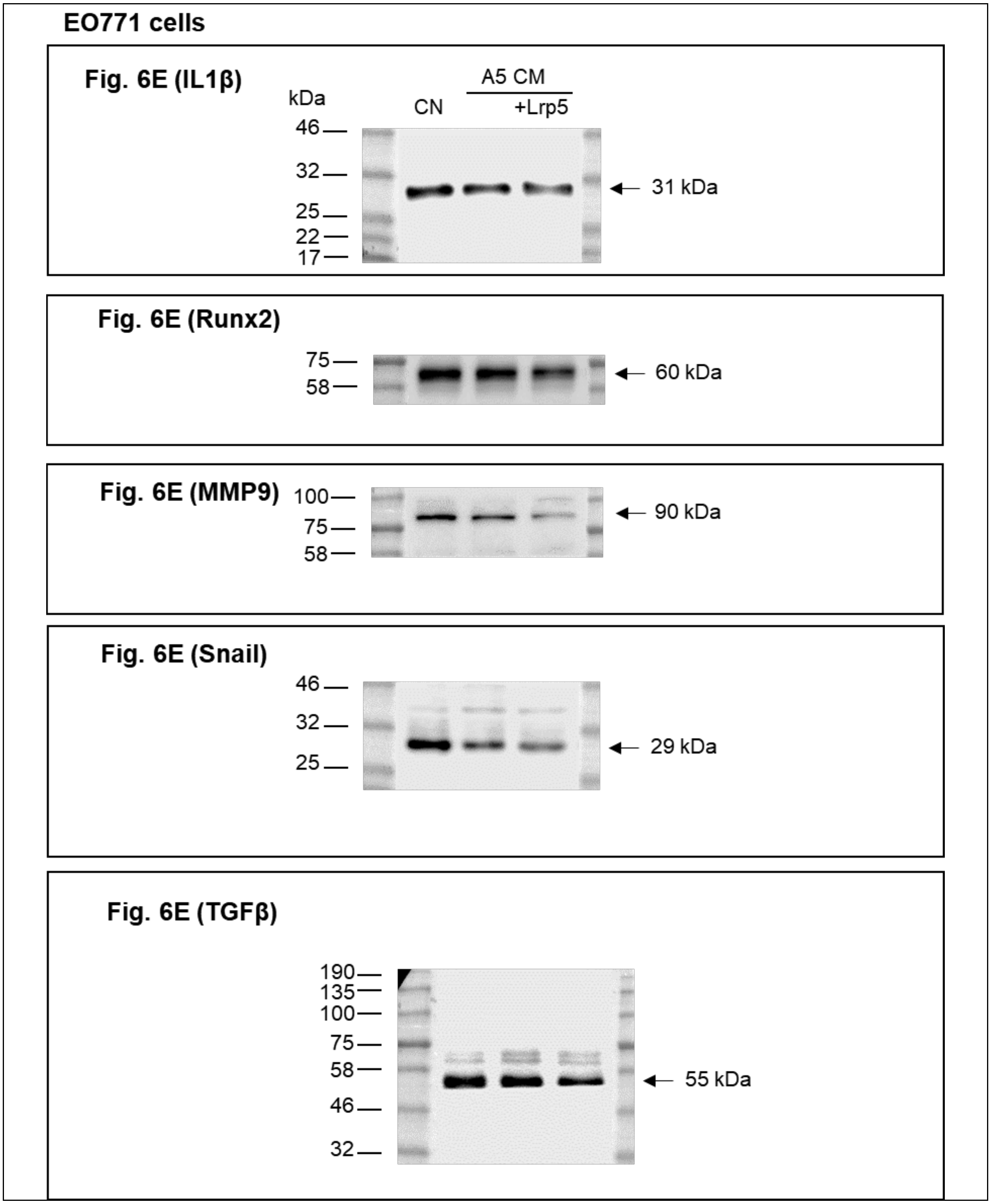
EO771 cells
Fig. 6E (IL1β)
kDa
A5 CM
CN
+Lrp5
46
32
25
22
17
31 kDa
Fig. 6E (Runx2)
75
58
60 kDa
Fig. 6E (MMP9)
100
75
58
90 kDa
Fig. 6E (Snail)
46
32
25
29 kDa
Fig. 6E (TGFβ)
190
135
100
75
58
46
32
55 kDa

Figure 6

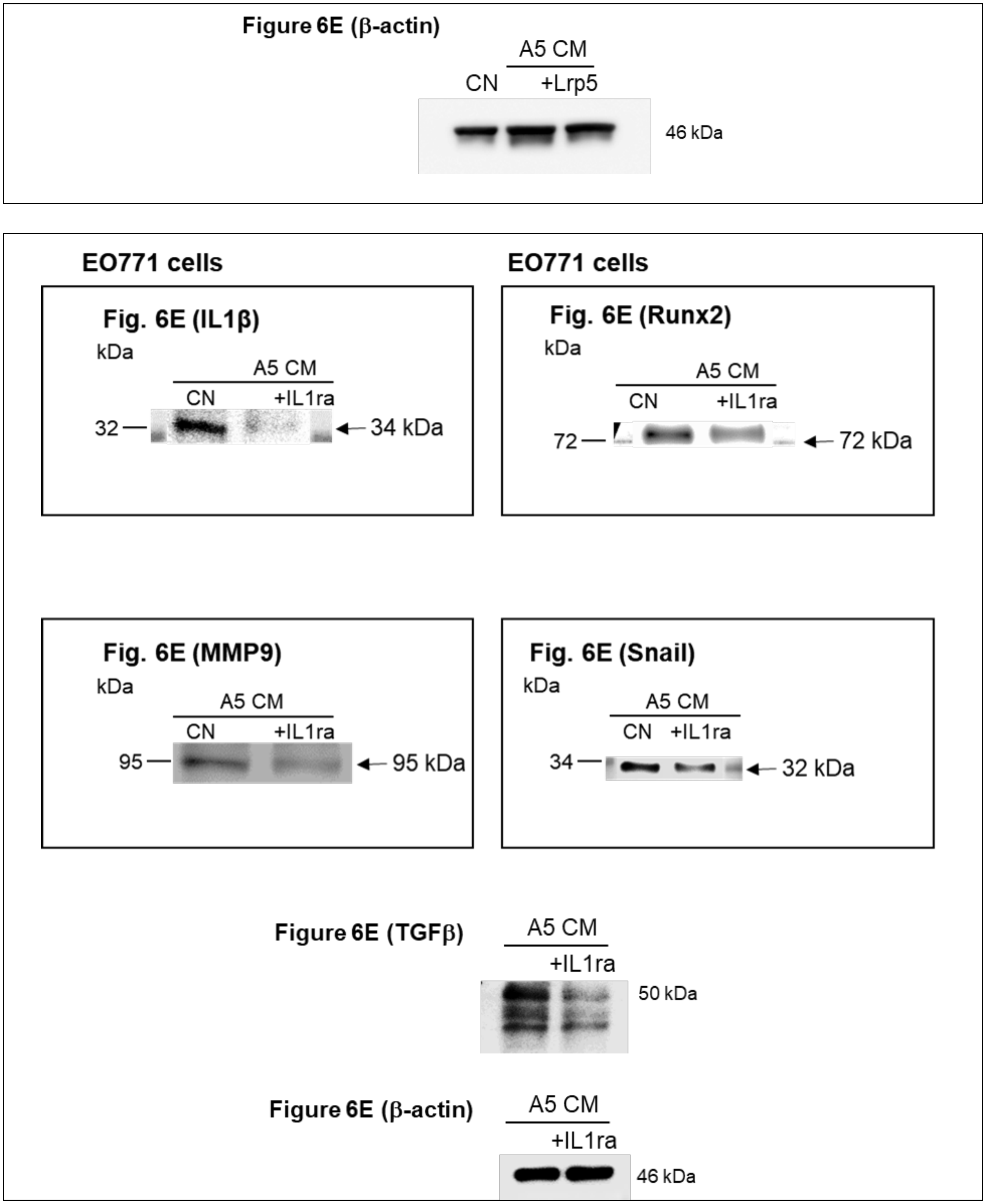

Figure 6E (β-actin)
A5 CM
CN
+Lrp5
46 kDa
EO771 cells
Fig. 6E (IL1β)
kDa
A5 CM
CN
+IL1ra
32
34 kDa
EO771 cells
Fig. 6E (Runx2)
kDa
A5 CM
CN
+IL1ra
72
72 kDa
Fig. 6E (MMP9)
kDa
A5 CM
CN
+IL1ra
95
95 kDa
Fig. 6E (Snail)
kDa
A5 CM
CN
+IL1ra
34
32 kDa
Figure 6E (TGFβ)
A5 CM
+IL1ra
50 kDa
Figure 6E (β-actin)
A5 CM
+IL1ra
46 kDa

Fig. 6E (IL1β)

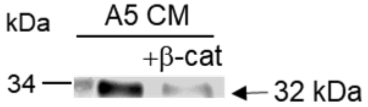

Fig. 6E (Runx2)

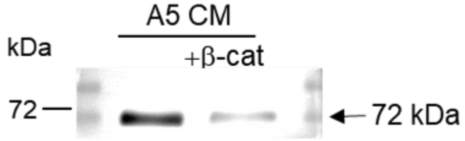

EO771 cells

Fig. 6E (TGFβ)

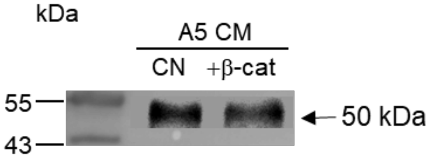

Fig. 6E (Snail)

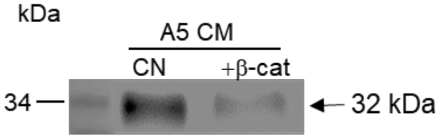

Figure 6E (MMP9)

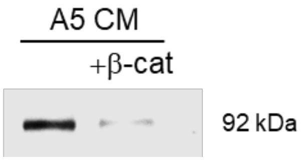

Figure 6E (β-actin)

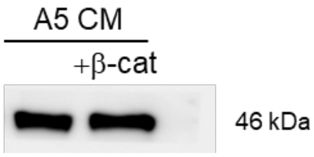

EO771 cells

Fig. 6F (p53)

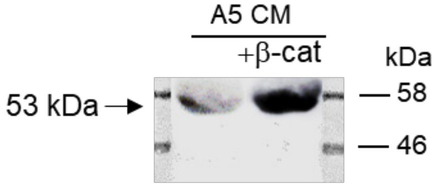

Fig. 6F (DSP)

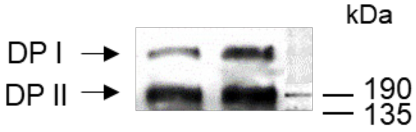

Figure 6

Figure 6F (TRAIL)

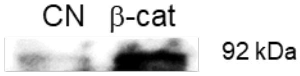

Figure 6F (LIMA1)

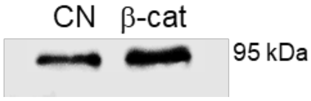

Figure 6F (CXCL1)

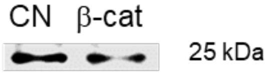

Figure 6F (CXCL5)

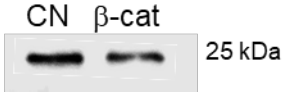

Figure 6F (β-actin)

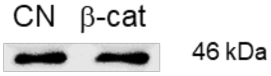

A5 cells

Fig. 6G (β-catenin)

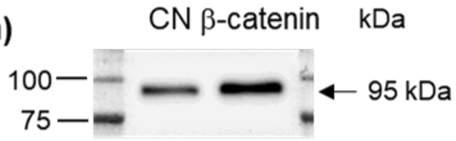

Figure 6F (β-actin)

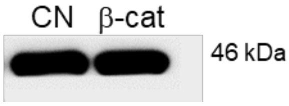

Figure 7

A5 CM

Fig. 7C (Ubc)

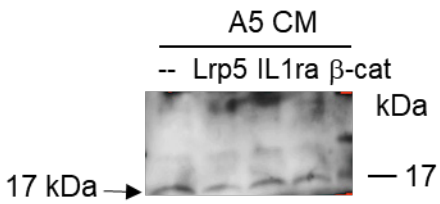

A5 CM

Fig. 7C (H4)

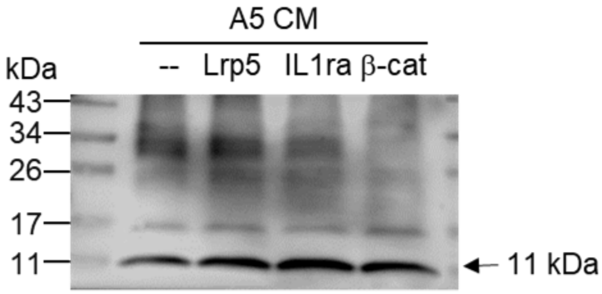

Figure 7C (β-actin)

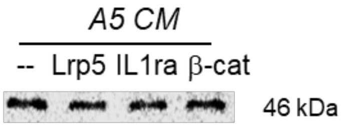

EO771 cells

Fig. 7I (Runx2)

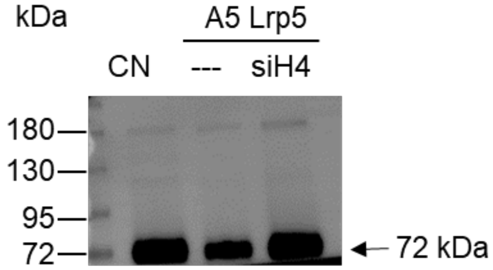

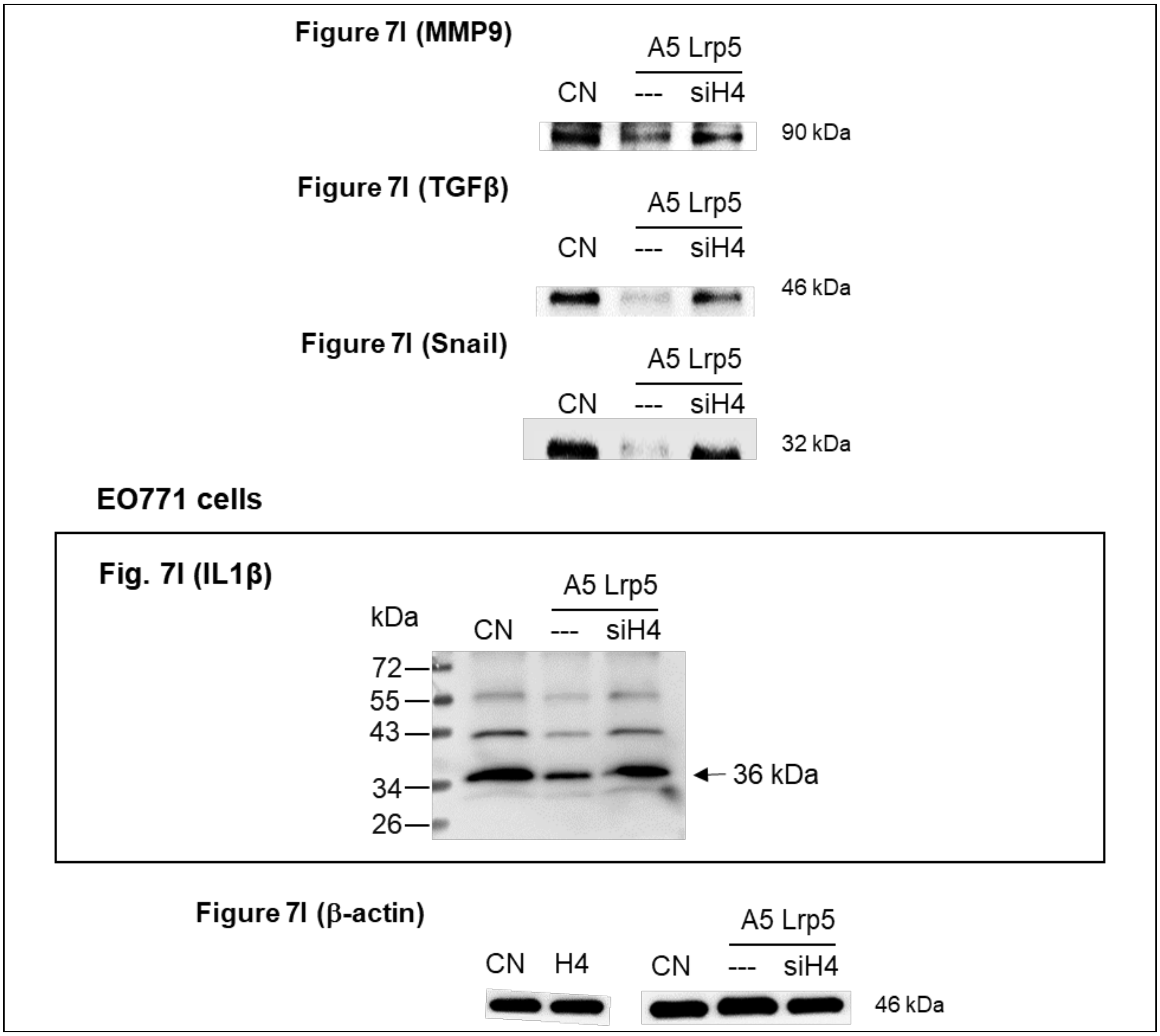
Figure 7I (MMP9)
A5 Lrp5
CN
---
siH4
90 kDa
Figure 7I (TGFβ)
A5 Lrp5
CN
---
siH4
46 kDa
Figure 7I (Snail)
A5 Lrp5
CN
---
siH4
32 kDa
EO771 cells
Fig. 7I (IL1β)
A5 Lrp5
kDa
CN
---
siH4
72
55
43
34
26
36 kDa
Figure 7I (β-actin)
A5 Lrp5
CN
H4
CN
---
siH4
46 kDa

Figure 7

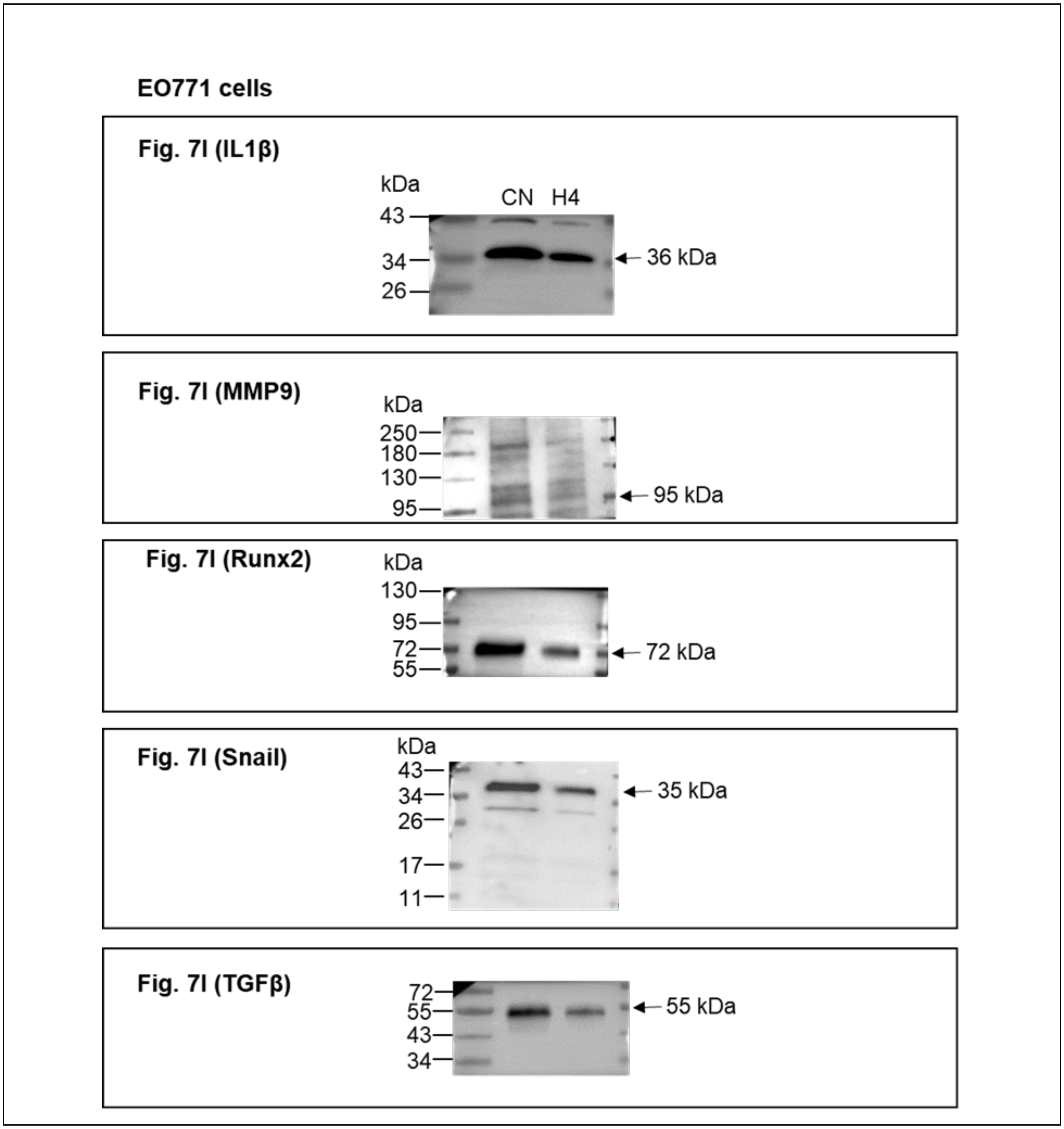
EO771 cells
Fig. 7I (IL1β)
kDa
CN H4
43
34
26
36 kDa
Fig. 7I (MMP9)
kDa
250
180
130
95
95 kDa
Fig. 7I (Runx2)
kDa
130
95
72
55
72 kDa
Fig. 7I (Snail)
kDa
43
34
26
17
11
35 kDa
Fig. 7I (TGFβ)
72
55
43
34
55 kDa

Figure 7

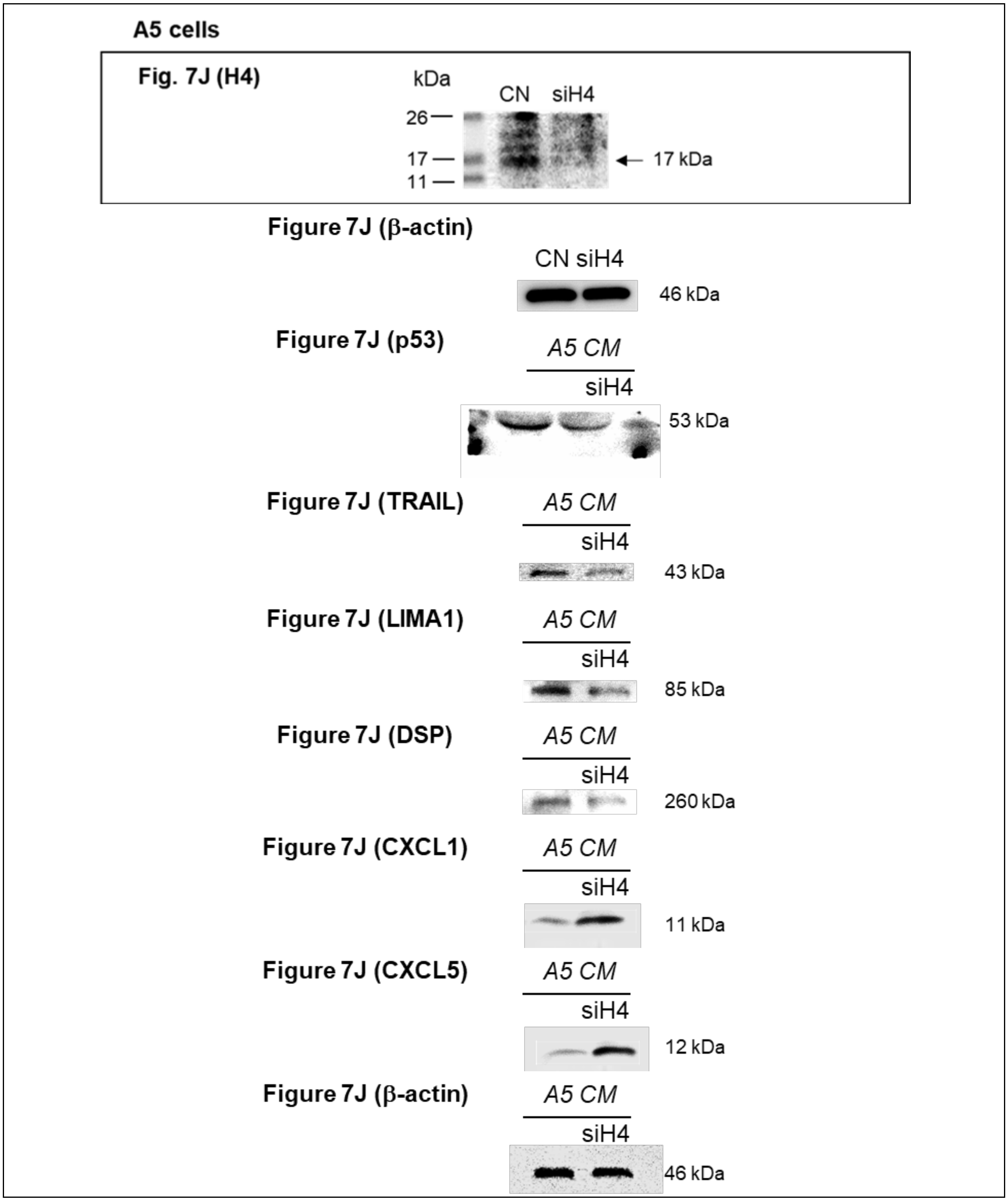
A5 cells
Fig. 7J (H4)
kDa
CN siH4
26
17
11
17 kDa
Figure 7J (β-actin)
CN siH4
46 kDa
Figure 7J (p53)
A5 CM
siH4
53 kDa
Figure 7J (TRAIL)
A5 CM
siH4
43 kDa
Figure 7J (LIMA1)
A5 CM
siH4
85 kDa
Figure 7J (DSP)
A5 CM
siH4
260 kDa
Figure 7J (CXCL1)
A5 CM
siH4
11 kDa
Figure 7J (CXCL5)
A5 CM
siH4
12 kDa
Figure 7J (β-actin)
A5 CM
siH4
46 kDa

Figure 8

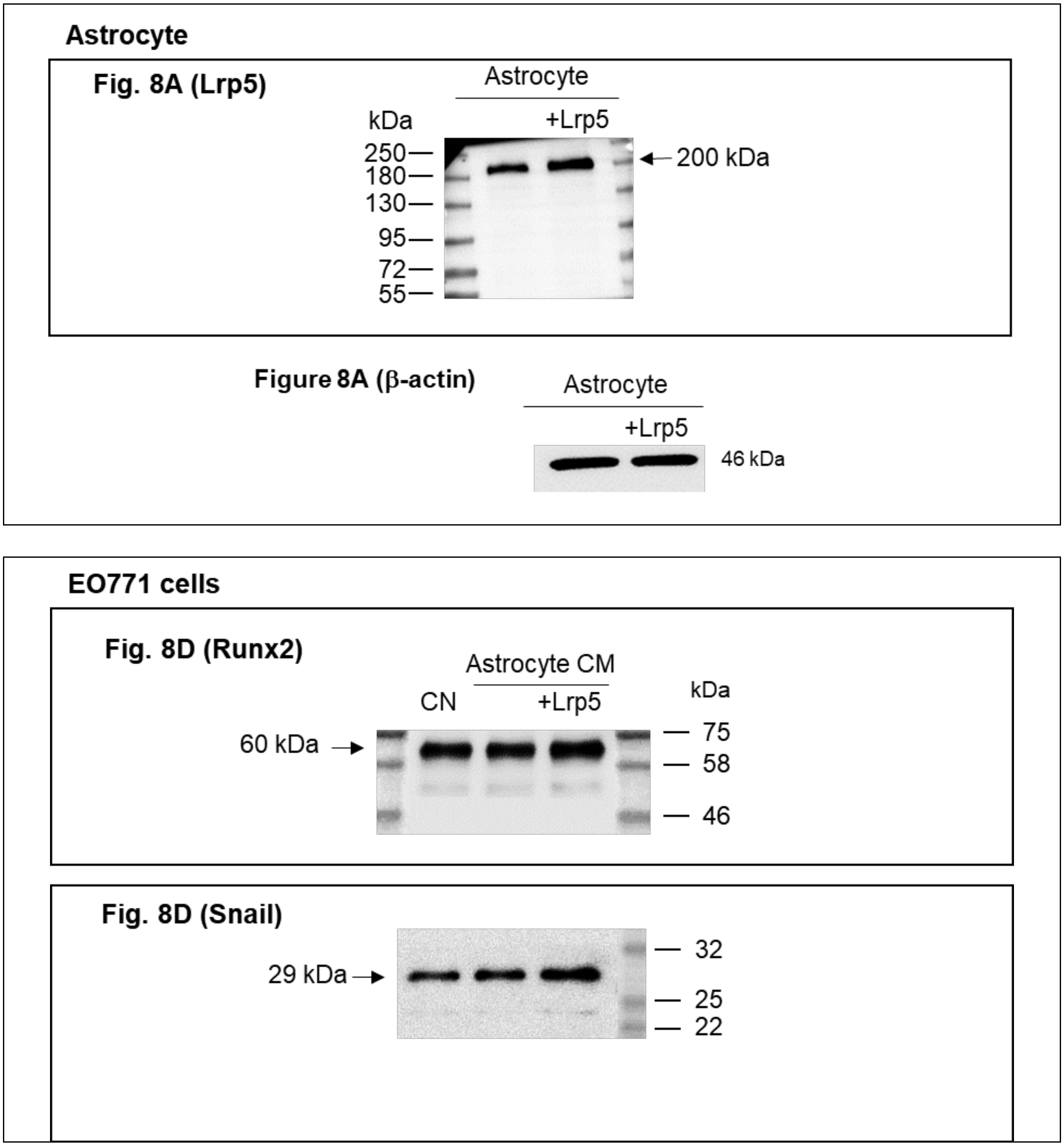
Astrocyte
Fig. 8A (Lrp5)
Astrocyte
kDa
+Lrp5
250
180
130
95
72
55
200 kDa
Figure 8A (β-actin)
Astrocyte
+Lrp5
46 kDa
EO771 cells
Fig. 8D (Runx2)
Astrocyte CM
CN
+Lrp5
kDa
60 kDa
75
58
46
Fig. 8D (Snail)
29 kDa
32
25
22

Figure 8

EO771 cells

Fig. 8D (MMP9)

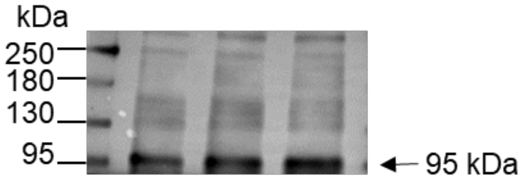

EO771 cells

Fig. 8D (MMP9)

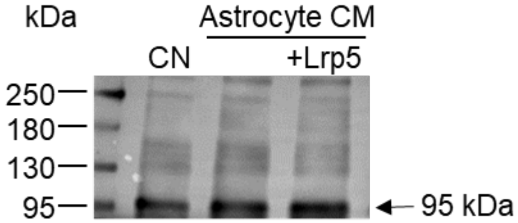

Figure 8D (β-actin)

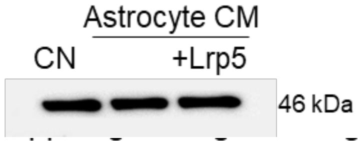

EO771 cells

Fig. 8F (N-cadherin)

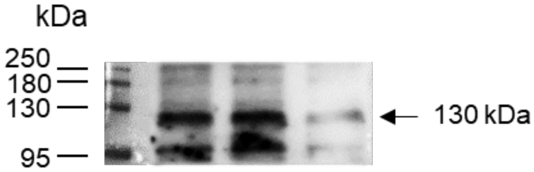

Figure 8F (β-actin)

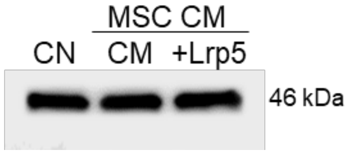

Figure 8

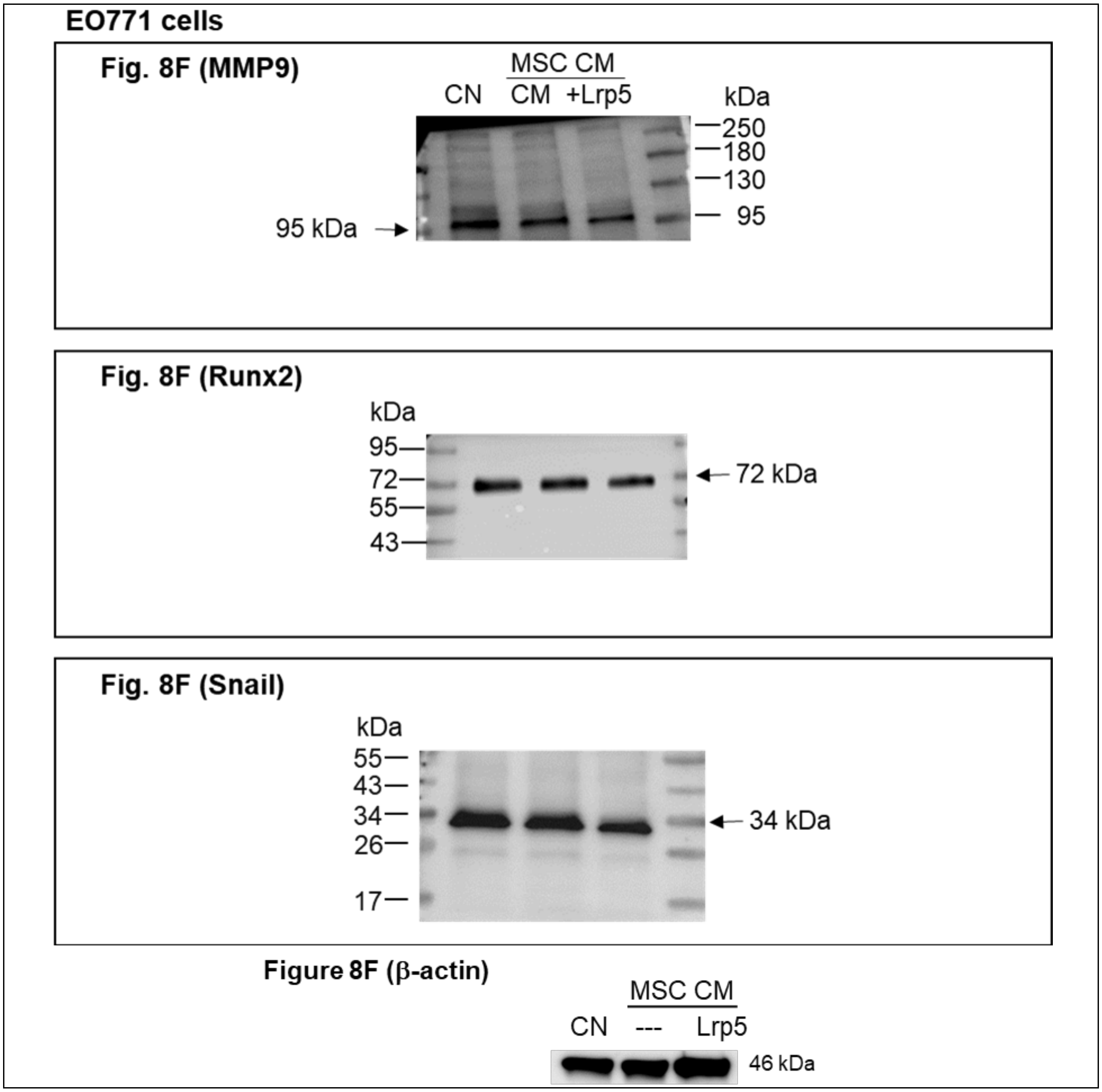
EO771 cells
Fig. 8F (MMP9)
MSC CM
CN
CM
+Lrp5
kDa
250
180
130
95
95 kDa
Fig. 8F (Runx2)
kDa
95
72
55
43
72 kDa
Fig. 8F (Snail)
kDa
55
43
34
26
17
34 kDa
Figure 8F (β-actin)
MSC CM
CN

Lrp5
46 kDa
