# Supplementary material for: Inhibition of the Growth of Breast Cancer-Associated Brain Tumors by the Osteocyte-Derived Conditioned Medium
Source: Cancers (Basel). 2021 Mar 3;13(5):1061. doi: 10.3390/cancers13051061 (PMC7959137; doi:10.3390/cancers13051061)
Supplement: Supplementary file 1 [file cancers-13-01061-s001.zip › Supplementary Materials/cancers-1088149-supplementary-for xml.docx]

Supplementary materials: Inhibition of the Growth of Breast Cancer-Associated Brain Tumors by the Osteocyte-Derived Conditioned Medium

Tomohiko Sano, Xun Sun, Yan Feng, Shengzhi Liu, Misato Hase, Yao Fan, Rongrong Zha, Di Wu, Uma K. Aryal, Bai-Yan Li, Akihiro Sudo and Hiroki Yokota

Estimation of the diffusion coefficient through the skull

Suppose that the two aqueous reservoirs are separated by a skull and the solute (albumin) in the first reservoir with the volume $V_{1}$ is transferred to the second reservoirs with the volume $V_{2}$. When the volume of the skull is negligible, the change in the solute in the first reservoir is approximated, using the quasi-steady-state flux, $N$:

$-V_{1}\frac{dc_{1}(t)}{dt}=A\cdot N$ Eq. (1)

$N=D\frac{c_{1}(t)-c_{2}(t)}{L}$ Eq. (2)

Where $t$ = time, and $c_{1}(t)$ and $c_{2}(t)$ = concentrations in the first and second reservoirs, respectively, $A$ = skull surface area, $D$ = diffusion coefficient through the skull, and $L$ = skull thickness. Since the decrease in the first reservoir is equivalent to the gain in the second reservoir:

$V_{1}\frac{dc_{1}(t)}{dt}=-A\cdot D\frac{c_{1}(t)-c_{2}(t)}{L}$ Eq. (3)

If the initial concentrations are $c_{1}\left( t=0 \right)=c_{0}$ and $c_{2}\left( t=0 \right)=0$, the concentration $c_{1}$ is predicted as a function of time:

$ln\frac{c_{1}\left( t \right)-\frac{V_{1}}{V_{1}+V_{2}}c_{0}}{\frac{V_{2}}{V_{1}+V_{2}}c_{0}}=-\frac{A\cdot D}{LV_{1}}\left( 1+\frac{V_{1}}{V_{2}} \right)t$ Eq. (4)

The diffusion coefficient of albumin in the skull was estimated as 3.30X10^-8^ cm^2^/s from the slope (0.7157) of the best-fit line of Eq. 4 in Figure 6B. Of note, $V_{1}$ = 265 μl, $V_{2}$ = 65 μl, $c_{0}$ = 10 μg/μl, $A$ = 188 mm^2^, and $L$ = 60 μm.

| 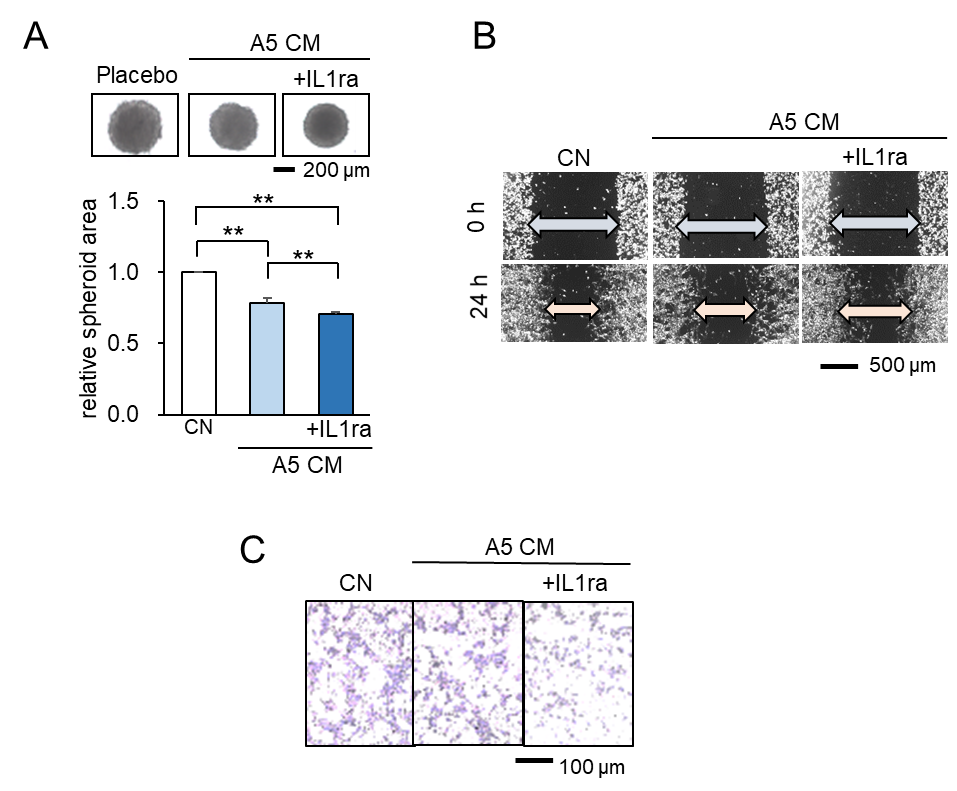 |
| --- |
| **Figure S1.** Anti-tumor effects of IL1ra overexpression in osteocytes. Of note, CN = control, and CM = conditioned medium. The double asterisks indicate *p* < 0.01. (A-C) Reduction in the size of tumor spheroids, migration in the scratch assay, and Transwell invasion in response to osteocyte-derived CM with and without the overexpression of IL1ra. |

| 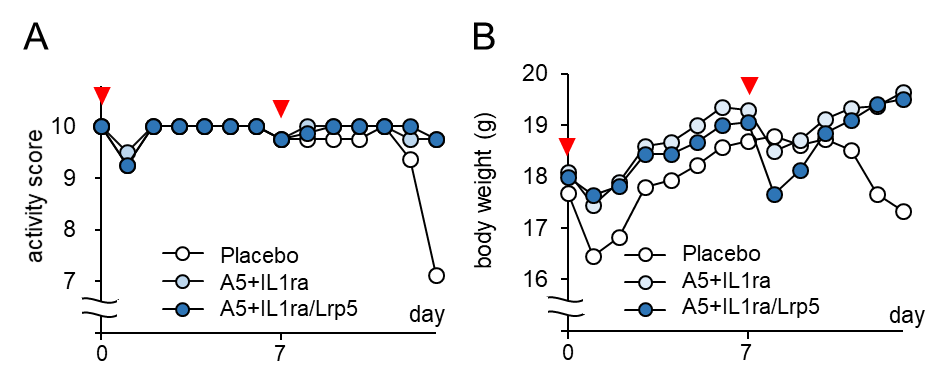 |
| --- |
| **Figure S2.** Suppressive effect of osteocytes, overexpressed with IL1ra, and/or Lrp5 in C57BL/6 mice (N = 8 per group). (A&B) Activity score and body weight for 2 weeks. The red arrowheads indicate the days osteocytes were injected. |

| 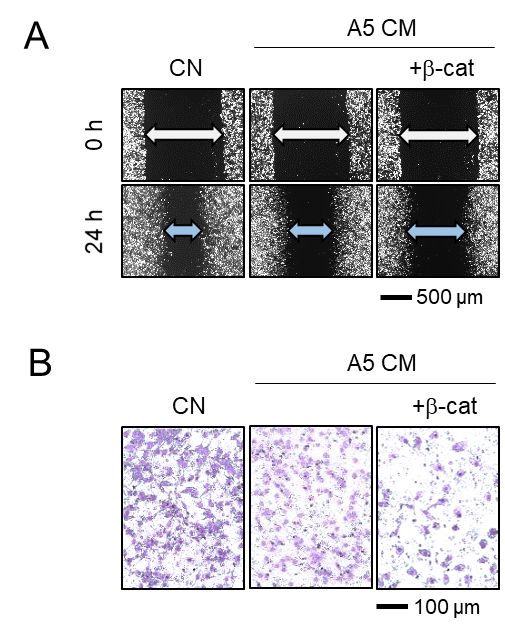 |
| --- |
| **Figure 3.** Cellular behavior of EO771 mammary tumor cells in response to β-catenin-overexpressing osteocyte-derived condition medium. Of note, CN = control, CM = conditioned medium, and β-cat = β-catenin. (A) Scratch-based migration assay. (B) Transwell invasion assay. |

| 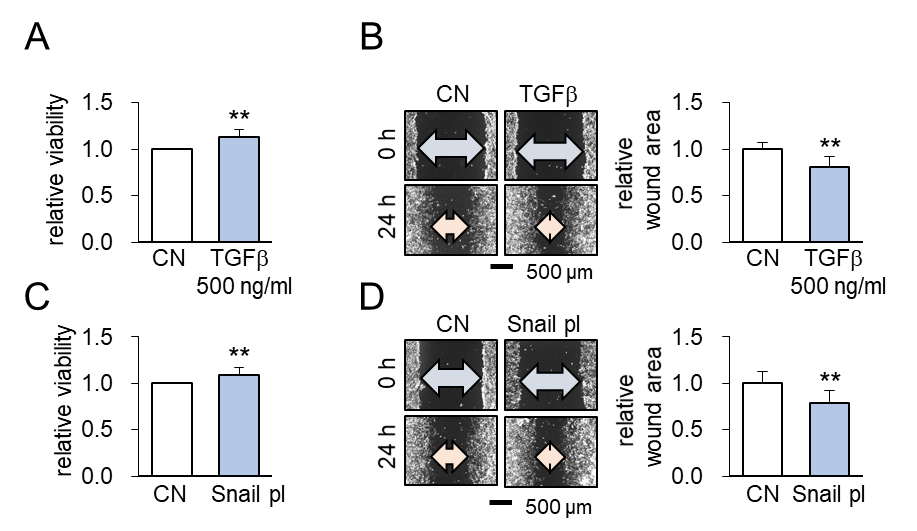 |
| --- |
| **Figure 4.** Cellular behavior of EO771 mammary tumor cells in response to TGFβ and Snail overexpression. Of note, CN = control, and snail pl = Snail overexpression by plasmid transfection. (A&B) Elevation of MTT-based viability and scratch-based motility by 500 ng/ml TGFβ for 1 day. (B) Elevation of MTT-based viability and scratch-based motility by the overexpression of Snail. |

| 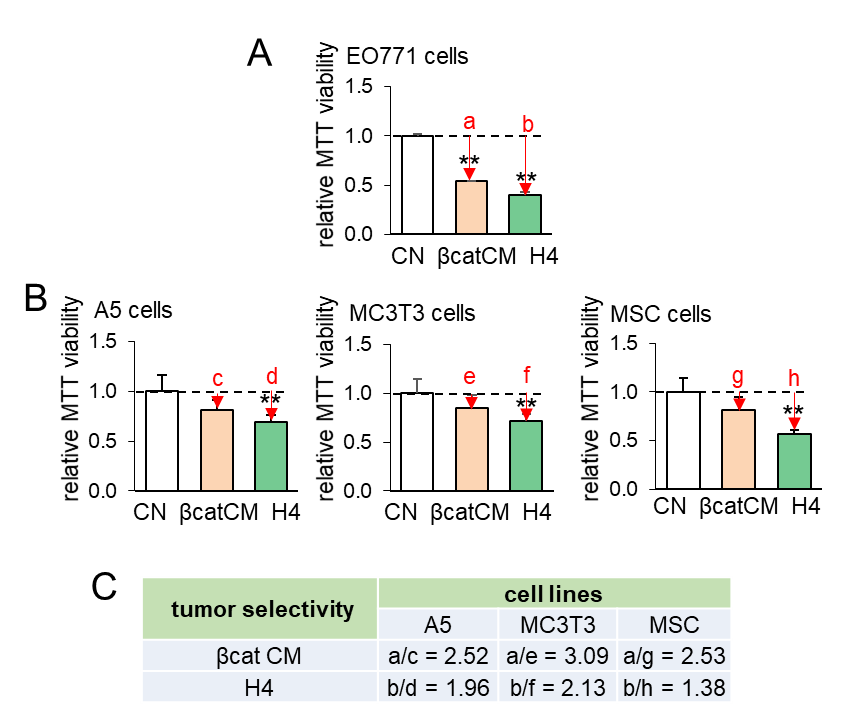 |
| --- |
| **Figure 5.** Effects of β-catenin-overexpressing osteocyte-derived condition medium and histone H4 on MTT-based viability of EO771 mammary tumor cells, MLO-A5 osteocytes, MC3T3 osteoblasts, and mesenchymal stem cells (MSCs). (A) MTT viability of EO771 cells. (B) MTT viability of non-tumor cells such as MLO-A5 osteocytes, MC3T3 osteoblasts, and MSCs. (C) Tumor selectivity of β-catenin-overexpressing osteocyte-derived condition medium and histone H4. The tumor selectivity is defined as (MTT reduction in tumor cells) divided by (MTT reduction in non-tumor cells). The tumor selectivity is above 1 in all cases, indicating that the inhibitory effects of these agents are selective to tumor cells. |

**Table S1.** Summary of the tumor-suppressing protein candidates (56 proteins) by the whole-genome proteomics analysis. Compared to the control medium, these proteins were enriched in the β-catenin-overexpressing osteocyte-derived condition medium.
